# Mixed-methods analysis of satisfaction during a 12-session mindfulness-based intervention for women with a substance use disorder and trauma symptomatology

**DOI:** 10.3389/fpsyg.2024.1359174

**Published:** 2024-06-06

**Authors:** Tara G. Bautista, Orrin D. Ware, Miracle A. Macias Burgos, Veronica D. Rivas, Yesenia Cruz-Carrillo, Alec Davidson, Mariia Mezhenska, Mariana Sanchez, Hortensia Amaro

**Affiliations:** ^1^Department of Psychological Sciences, Northern Arizona University, Flagstaff, AZ, United States; ^2^School of Social Work, University of North Carolina at Chapel Hill, Chapel Hill, NC, United States; ^3^Department of Psychology, Arizona State University, Tempe, AZ, United States; ^4^Robert Stempel College of Public Health and Social Work, Florida International University, Miami, FL, United States

**Keywords:** satisfaction, trauma, residential treatment, mindfulness, substance use disorder, women

## Abstract

Satisfaction with an intervention influences the uptake of behavior changes and the long-term efficacy of the intervention. Therefore, it is crucial to assess satisfaction by participant profile when creating and adapting behavior interventions for minoritized populations. Qualitative and quantitative data on participant trauma symptom severity and intervention satisfaction were collected through self-report surveys from 54 women. The sample was 59.3% Hispanic, with an average age of 33.21 (SD = 10.42), who were in residential treatment for substance use disorders (SUDs) and participated in a 12-session mindfulness-based intervention. Qualitative responses were coded using thematic analysis, and an integrative mixed-methods approach was used to compare qualitative theme frequency between high-trauma (*N* = 28) and low-trauma (*N* = 26) groups at session 2 and session 11. High- and low-trauma groups were determined by interquartile ranges (bottom 25% = low; top 75% = high). In session 2, the low-trauma group reported significantly higher satisfaction (*M* = 4.20, SD = 0.55) than the high-trauma group (*M* = 3.77, SD = 0.89); *t*(43) = 1.90, *p* = 0.03. In session 11, there was no significant difference between groups. The mixed-methods analysis revealed that “trouble focusing” appeared more frequently in the high-trauma group than in the low-trauma group during session 2, but the theme was not present in either group at session 11, suggesting that this might pose an initial barrier for individuals with high trauma but subsides as the intervention progresses. This speaks to the importance of retention strategies tailored for participants with SUDs and high trauma while they adjust to the intervention. Assessing initial challenges with satisfaction may help facilitators intervene to increase participant satisfaction.

## Introduction

Substance use disorders (SUDs) and the experience of traumatic events throughout one’s life are closely interconnected. This connection is so strong many individuals with a SUD also suffer from a trauma- and stressor-related disorder (Pilowsky et al., 2009; Enoch, 2011; Goldstein et al., 2016; Grant et al., 2016; Verplaetse et al., 2018; Rosic et al., 2021; Ware et al., 2023). For example, having a SUD is associated with a 1.5 risk of having PTSD compared to individuals who have not had a SUD in their lifetime (Goldstein et al., 2016). Furthermore, evidence suggests that many individuals use substances to cope with emotions or thoughts associated with previously experiencing traumatic events, which increases their risk of developing a SUD (Thornton et al., 2012; Russell et al., 2017; Hawn et al., 2020). Despite the association between trauma and SUD, not all SUD treatment providers offer trauma-specific treatment services (Spivak et al., 2022), highlighting the potential for suboptimal treatment for these frequently co-occurring conditions. A study that examined all known SUD treatment providers in 2019 found that approximately 43% offered trauma-specific treatment services (Spivak et al., 2022). Despite less than half of all SUD treatments offering trauma-informed care, those with trauma-informed care have been found to be more effective at increasing retention and reducing relapse (Bartholow and Huffman, 2023). Therefore, addressing trauma during SUD treatment may enhance post-treatment outcomes.

The experiences of trauma exposure vary by key sociodemographic characteristics, such as gender. Women are particularly vulnerable to experiencing traumatic events as studies have found that women are more likely to have PTSD than men (excluding veterans and members of the armed forces) (Ditlevsen and Elklit, 2010; Ditlevsen and Elklit, 2012; Gradus, 2017; Spottswood et al., 2017). Women with PTSD may engage in a higher frequency of substance use than their male counterparts (Mitra et al., 2021). Moreover, women with a SUD are more likely to have experienced traumatic events (Tripodi and Pettus-Davis, 2013; Ullman et al., 2013; Devries et al., 2014; Stein et al., 2017; Cafferky et al., 2018; Muchimba, 2020). It is recommended that any SUD treatment addressing the specific needs of women incorporate a trauma-informed approach to simultaneously address potential histories of trauma. Despite the benefits of SUD treatment (e.g., longer periods of reduced substance use and lower criminal justice involvement), it is estimated that only 50% of individuals with co-occurring SUDs and mental health disorders receive any treatment for either disorder (Administration S.A.A.M.H.S, 2023).

Once an individual enters SUD treatment, longer treatment retention is associated with positive outcomes such as reduced substance use after discharge (Hser et al., 2004; Daigre et al., 2021). Persons who are satisfied with their treatment may be more likely to have longer treatment retention. Compared to individuals receiving outpatient SUD treatment, those receiving residential SUD treatment may be particularly vulnerable to leaving treatment prematurely. Residential treatment is often prescribed for persons with lower environmental support and an increased risk of withdrawal or returning to previous patterns of substance use (Mee-Lee et al., 2013). Therefore, treatment satisfaction is especially important for individuals engaged in residential SUD treatment as they are particularly vulnerable and often lack stable support in their environment should they leave treatment prematurely. Residential SUD treatment interventions for women that are trauma-informed *and* in which the individuals feel satisfied would be considered superior to interventions being trauma-informed only without satisfaction and vice versa (e.g., satisfaction only without being trauma-informed).

Mindfulness-based interventions (MBIs) have shown promising results in treating SUD and trauma individually (Gallegos et al., 2017; Hopwood and Schutte, 2017; Félix-Junior et al., 2022). There is also a growing body of evidence supporting the use of MBIs to treat both SUD and trauma concurrently (Amaro and Black, 2021; Somohano et al., 2022). Over 15 years ago, Vallejo and Amaro (2009) recommended all MBIs be minimally trauma-informed, but preferably trauma-responsive, due to the high overlap of common sequelae of addiction and trauma. This is especially important for MBIs to consider because mindfulness practices often involve connections between the mind and body, and there are parts of the body that are more sensitive and likely to trigger traumatic memories (Vallejo and Amaro, 2009). MBIs have also shown greater acceptability and efficacy among women from racial and ethnic minoritized communities (Witkiewitz et al., 2013). This is of particular importance as racial and ethnic minorities are often more likely to leave SUD treatment prematurely, especially those with a lower socioeconomic status (Saloner and Cook, 2013; Stahler et al., 2016). The promising evidence of MBI to treat trauma and SUD and the acceptability of MBI among racial and ethnic minorities highlight the strong potential of this evidence among some of the most vulnerable populations receiving SUD treatment.

Moment-by-Moment in Women’s Recovery (MMWR) is an MBI adapted for low-income, ethnoracially diverse women with low education and a history of trauma. In a previous study published in MMWR, using the same sample as the present study, it was found that baseline trauma symptom severity was positively associated with the uptake of mindfulness practices by session 3 of the 12-session program (Bautista and Amaro, 2023). This may suggest that women with high-trauma symptom severity at baseline were using more mindfulness practices as a response to both the trauma symptoms and the SUD symptoms.

This secondary data analysis study was inspired by clinical observations made by facilitators during the intervention. They noted that participants with high-trauma symptom severity at the beginning of the intervention tended to report more complaints and lower satisfaction with the intervention in the first few sessions. These observations were discussed during research team meetings, prompting the current study. The purpose of the present study was to examine differences (both qualitative and quantitative) in the satisfaction of MMWR between women with high-trauma symptom severity and women with low-trauma symptom severity. We assessed qualitative narratives of participant satisfaction at session 2 and session 11 of the 12-session MMWR program and how the frequency of themes differed by participant trauma symptom severity scores. We hypothesized that:

1. Satisfaction scores at session 11 will be higher than satisfaction scores at session 2 for both the high- and low-trauma symptom severity groups.
2. Satisfaction scores will be higher among those with low-trauma symptom severity than those with high-trauma symptom severity at session 2 and at session 11.

## Methods

### Participants

A subsample of 54 participants was selected (based on trauma symptom severity score) from a sample of 100 adult women who were randomized to the Moment-by-Moment in Women’s Recovery (MMWR) intervention conditions (Stahler et al., 2016). The participants that were randomized to the education control group were not included in the present study because they did not have a measure of satisfaction with the MMWR, which is the focus of this paper. Black and Amaro (2019) found no significant difference in satisfaction scores between the intervention and educational control group (Black and Amaro, 2019). The University of Southern California Institutional Review Board approved this study (UP-14-00391). All persons gave informed consent prior to their inclusion in the study. All participants were clients at a residential SUD treatment and clinically diagnosed with SUD based on the Diagnostic and Statistical Manual of Mental Disorders, Fifth Edition (American Psychiatric Association, 2013). The residential treatment facility was publicly funded and had a capacity for up to 110 women and their children. The facility offered childcare, mental health, and SUD diagnosis and treatment, individual and group education and counseling, relapse prevention training, health and wellness activities including nutrition education, and 12-step meetings. The inclusion criteria comprised individuals who were clients at the residential treatment study site, female, aged 18–65 years, diagnosed with SUD, fluent in English, and agreed to participate in the study. Participants were excluded if they were unable to understand and sign the informed consent form, had cognitive impairments, untreated psychiatric disorders, were more than 6 months pregnant, enrolled in another study, or were not willing to sign a HIPAA form or be audio recorded during interviews and intervention sessions. The subsample was selected to represent those with the highest and lowest scores of trauma symptom severity based on their interquartile ranges (see PTSD Symptom Scale description below). The trauma symptom severity score for the full sample was *M* = 16.23 (SD = 11.94), the low-trauma symptom severity group (*N* = 26) were participants in the bottom quartile (25%), and the high-trauma symptom severity group (*N* = 28) were participants in the top quartile (75%). Table 1 provides participant characteristic information for the subsample used in the present study.

**Table 1:** Demographics of 54 Women in SUD Tx.

Variable	*M* (SD) or *N* (%)		Sig.
	Low PTSS (*N* = 26)	High PTSS (*N* = 28)	
Age	32.99 (10.11)	33.42 (10.89)	NS
Years of education	11.46 (1.75)	11.75 (2.56)	NS
Race/ethnicity			
Hispanic	15 (57.7%)	17 (60.7%)	
Non-Hispanic Black	7 (26.9%)	3 (10.7%)	
Non-Hispanic white	4 (15.4%)	7 (25.0%)	NS
Mandated to treatment	22 (84.6%)	21 (75%)	NS
Past 8-month substance use (yes/no)			
Alcohol to intoxication (yes)	18 (69.2%)	23 (82.1%)	NS
Amphetamines (yes)	4 (15.4%)	5 (17.9%)	NS
Methamphetamine (yes)	24 (92.3%)	23 (82.1%)	NS
Barbiturates (yes)	1 (3.8%)	3 (10.7%)	NS
Powder cocaine (yes)	15 (57.7%)	21 (75%)	NS
Crack/rock cocaine (yes)	13 (50.0%)	8 (28.6%)	NS
Heroin (yes)	5 (19.2%)	6 (21.4%)	NS
Total number of sessions attended	8.77 (3.88)	9.64 (3.01)	NS
Session 2 satisfaction	4.20 (0.55)	3.77 (0.89)	*p* = 0.03
Session 11 satisfaction	4.53 (0.42)	4.26 (0.62)	NS

### Intervention

The *Moment-by-Moment in Women’s Recovery* (MMWR) program was conducted in a group format as a part of residential treatment, with sessions held twice a week for a total of 12 sessions spanning 6 weeks. Each session lasted for 80 min. The MMWR facilitators were trained in both mindfulness-based stress reduction (MBSR) and MMWR. There was also a trained on-site master’s-level clinician with experience in SUDs present during the sessions. The facilitators used an instructional manual with standardized lesson plans (Amaro and Black, 2017; Black and Amaro, 2019).

### Measures

#### Post-traumatic stress disorder (PTSD) symptom severity

The PTSD Symptom Scale (PTSS)—Self Report, composed of 17 items (rated from 0 = not at all to 3 = almost always), was administered at baseline. A sample item is “How often have you been bothered by having bad dreams or nightmares about the traumatic events?” This scale measures the frequency of reexperiencing, avoidance, and arousal symptoms related to trauma exposure over the past 30 days. Reliability and validity have been shown for assessing PTSD symptoms experienced by the participants in the last month (Foa et al., 2005). For the current sample, the total scale score was used, and Cronbach’s alpha was *α* = 0.93.

#### Satisfaction survey

Satisfaction data were collected at the end of session 2 and session 11 of the 12-session program. The goal of assessing satisfaction at the beginning and the end of the intervention was to evaluate potential changes in satisfaction with more exposure to the intervention. Session 2 and session 11 were selected to give the participants some exposure (two sessions) for their first assessment and to complete the last rating at session 11 to give the participants maximum exposure to the intervention without interfering with the post-intervention assessments planned for the end of session 12. The quantitative satisfaction items consisted of 17 items rated from 1 (not at all) to 5 = (very much), with high scores indicating higher satisfaction; then, a mean score was calculated. The items assessed various aspects of satisfaction: session content, skills learned, perceived usefulness, and importance for recovery. For the current sample, Cronbach’s alpha was *α* = 0.95. The satisfaction survey also included qualitative questions that asked, “Please tell us what you liked most about the group?” and “Please tell us what you liked least about the group or what could be improved?”

### Data analytic plan

#### Quantitative

The high-trauma symptom severity group included participants who were in the 75% percentile, and the low-trauma symptom severity group included participants who were in the 25% percentile. Extreme groups analysis allows for comparison that can be useful in mixed-methods research. The qualitative themes may be too nuanced to see gradient differences on a continuous variable, but by grouping them into high and low categories, we can more easily capture meaningful differences between groups. The groups were divided to display satisfaction scores at session 2 for those with high and low baseline trauma symptom severity and satisfaction scores at session 11 for those with high and low baseline trauma symptom severity, making a total of four groups (session 2 low trauma, session 2 high trauma, session 11 low trauma, and session 11 high trauma). Satisfaction total score means were compared for individuals with high-trauma symptom severity and individuals with low-trauma symptom severity at session 2 and session 11. Independent-samples *t*-tests were conducted to test mean differences in satisfaction scores between participants with high- or low-trauma symptom severity at session 2 and separately for session 11. Paired-samples *t*-tests were used to test for mean differences in satisfaction scores from session 2 to session 11 within each group separately for high- and low-trauma symptom severity. One-tailed *t*-tests were justified based on theoretical support for the hypothesized direction of effect.

#### Qualitative

For the qualitative analysis, the constant comparison method was used to iteratively identify and categorize codes that emerged within and across questions (Charmaz, 2014). First, all responses were read, and then, the content of the answers was coded by two independent raters. Any disagreements in coding were discussed between the two independent raters, and if they could not reconcile the code, a third rater reviewed the case and then met with the two raters to discuss the most accurate code. Codes with common underlying meanings were grouped together to create themes. Themes were constantly refined and reordered during the process of thematic analysis (Braun and Clarke, 2006). The final themes were reviewed and agreed on by six authors (T.B., M.A.M.B., Y.C.C., V.R., A.C.D., and M.M.). To acknowledge subtle nuanced differences in responses, we created multiple individual codes and then grouped them into themes. For example, if someone mentioned they most liked the “awareness of emotions” while someone else mentioned that they most liked the “awareness of cravings,” we created two separate codes to capture the difference in meaning between these types of awareness, but we grouped them under the theme of “awareness” to capture the commonality and compare themes across groups.

#### Mixed methods

The data were collected with a convergent mixed-methods design. To integrate the qualitative and quantitative findings, the qualitative themes were presented by quantitative ratings of trauma symptom severity. We used a joint display to compare the qualitative theme with quantitative ratings of trauma symptom severity.

## Results

### Quantitative

Due to the small sample size, quantitative analyses were limited to bivariate assessments. To assess for differences between the low PTSS group and the high PTSS group, we conducted *t*-tests on the continuous variables to assess mean differences and chi-square tests to assess differences between groups on categorical variables. There was a significant difference in satisfaction scores at session 2 for the low-trauma symptom severity group (*M* = 4.20, SD = 0.55) compared to the high-trauma severity group [*M* = 3.77, SD = 0.89; *t*(43) = 1.90, *p* = 0.03, one-tailed]. There was no significant difference in satisfaction scores at session 11 for the low-trauma symptom severity group (*M* = 4.53, SD = 0.42) compared to the high-trauma symptom severity group [*M* = 4.26, SD = 0.62; *t*(34) = 1.54, *p* = 0.07, one-tailed]. There were no other significant differences between groups on any other variables. A paired-samples *t*-test was conducted to test if the increases in satisfaction scores from session 2 to session 11 were significant within each group. The results for the low-trauma symptom severity group indicated a significant difference between the satisfaction score at session 2 (*M* = 4.36; SD = 0.42) and the satisfaction score at session 11 (*M* = 4.57; SD = 0.39); [*t*(13) = −2.42, *p* = 0.02, one-tailed]. The results for the high-trauma symptom severity group also indicated a significant difference between the satisfaction score at session 2 (*M* = 3.55; SD = 0.95) and the satisfaction score at session 11 (*M* = 4.24; SD = 0.63); [*t*(16) = −3.13, *p* < 0.01, one-tailed]. Note that the means for the paired-samples *t*-test are marginally different in rounding than the means for the independent samples *t*-test due to very few participants not having a quantitative score for both sessions to be used in the paired analysis. For the low-trauma symptom severity group, there were five participants missing scores for satisfaction at session 2 and nine participants missing satisfaction scores for session 11. For the high-trauma symptom severity group, there were four participants missing satisfaction scores for session 2 and nine participants missing satisfaction scores for session 11. There were 31 participants with satisfaction scores for session 2 and session 11.

### Qualitative

**Figure 1:**
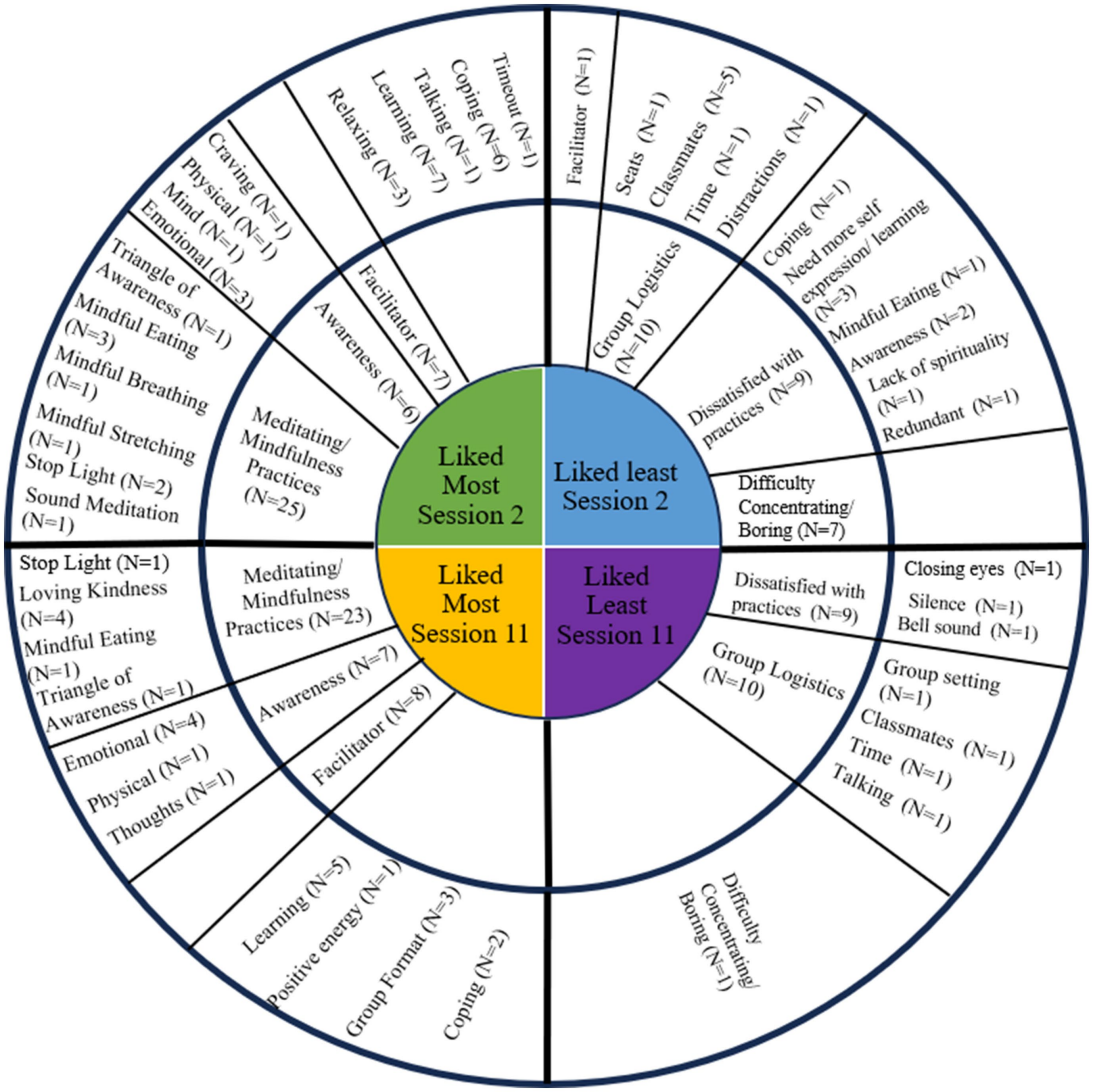
The wheel is divided into four quadrants with codes and themes from each satisfaction question. The innermost circle displays the satisfaction question the codes and themes resulted from, the middle circle displays the themes, and the outer circle displays the codes. If there were codes that did not result in a common theme, they are listed on the outer circle without a corresponding theme.

The thematic analysis resulted in 16 codes and 3 major themes for what participants liked most at session 2; the major themes were “meditating/mindfulness practices,” “awareness,” and “facilitator.” There were 12 codes and 3 major themes for what participants liked most in session 11; the major themes were the same as in session 2. There were twelve codes and three major themes for what participants liked least at session 2; the major themes were “group logistics,” “dissatisfied with practices,” and “difficulty concentrating/boring.” There were eight codes and two major themes for what participants liked least at session 11; the major themes were “group logistics” and “dissatisfied with practices.” All codes and themes are presented in Figure 1.

### Mixed methods

The integration of the quantitative findings indicated that participants with high-trauma symptom severity reported lower satisfaction with the intervention at both session 2 and session 11. Qualitative findings highlighted participants’ preferences for facilitators, practices, and awareness and least liked group logistics and difficulty concentrating led to the investigation of themes by trauma symptom severity group. The qualitative findings highlighted that the participants liked the facilitators, practices, and awareness and did not like the group logistics and reported difficulty concentrating. These findings led to the mixed methods investigation of themes separated by trauma symptom severity group. The results of the mixed-methods analysis show that participants with high-trauma symptom severity mentioned the facilitator more frequently (compared to those with low-trauma symptom severity) as what they liked most at session 2 and at session 11. We also found that participants with high-trauma symptom severity (compared to those with low-trauma symptom severity) more frequently reported trouble focusing/being bored as what they liked least about session 2, but trouble focusing/bored was not as prominent for either group at session 11. The most common themes for what participants liked most about each session, divided by high- and low-trauma symptom severity groups, are presented in Table 2. Additionally, the most common themes for what participants liked least about each session, divided by high- and low-trauma symptom severity groups, are presented in Table 3.

**Table 2:** Liked most about MMWR.

Group	**Session 2**	**Session 11**
**Low PTSS (N = 26)** **25th percentile**	Mindfulness practices (N = 14) Awareness (N = 4) Facilitator (N = 3)	Mindfulness practices (N = 12) Awareness (N = 3) Facilitator (N = 2)
**High PTSS (N = 28)** **75th percentile**	Mindfulness practices (N = 12) Learning (N = 5) Facilitator (N = 4)	Mindfulness practices (N = 11) Awareness (N = 4) Facilitator (N = 6)

**Table 3:** Liked least about MMWR.

	**Session 2**	**Session 11**
Low PTSS (N = 26) 25th percentile	Group logistics (N = 5) Trouble focusing/bored (N = 1)	Group logistics (N = 4) Mindfulness practices (N = 4)
High PTSS (N = 28) 75th percentile	Trouble focusing/bored (N = 6) Group logistics (N = 5)	Mindfulness practices (N = 6) Group logistics (N = 6)

## Discussion

This study investigated patterns in satisfaction across trauma symptom severity profiles and over time in a mindfulness-based intervention designed for diverse women with a history of trauma. We found participants with high-trauma symptom severity had lower satisfaction at session 2, but comparable satisfaction at session 11 as compared to participants with low-trauma symptom severity. We also found that satisfaction increased from session 2 to session 11 for individuals with high-trauma symptom severity and low-trauma symptom severity. These findings suggest that while participants with high-trauma symptom severity report lower satisfaction than participants with low-trauma symptom severity at the start of the intervention, their satisfaction with the intervention increases over time with greater exposure to the intervention.

The mixed-methods findings provide greater context to the quantitative findings. Here, we see that participants with high-trauma symptom severity reported having difficulty focusing during session 2, but not during session 11. This finding suggests that while participants with high-trauma symptom severity have difficulty focusing during session 2, with greater exposure to mindfulness practices throughout the intervention, their difficulty with focusing subsides. We also found that across both sessions, participants in the high-trauma symptom severity group reported the facilitator as the factor they liked the most during the intervention. This suggests that the facilitator may play a more impactful role in participant satisfaction among participants with high-trauma symptom severity, and the intervention facilitator may need additional training in trauma-informed care when delivering mindfulness-based interventions.

The MMWR program has been found to be effective at delaying the time to first cannabis use post-intervention (as compared to the control group), and within the MMWR group, greater attendance was shown to be positively associated with greater length of time to alcohol intoxication following treatment, fewer days of alcohol intoxication, and improvement in mindfulness skills (Amaro and Black, 2021). It is also worth highlighting that the MMWR program had an impressive attendance rate with 74% of participants in the MMWR group completing at least 9 of the 12 sessions and over 90% of participants completing their post-intervention self-report surveys (Black and Amaro, 2019). With MMWR class attendance (a proxy for dose response) being a protective factor of alcohol intoxication, there is a need for further research to examine what promotes greater class attendance or greater exposure to the MMWR curriculum. Due to MMWR being delivered in residential treatment, attendance may not be completely within the participants’ control; therefore, studying satisfaction and uptake of mindfulness practice in addition to class attendance may give a more comprehensive view of dose response and intervention acceptability (Bautista et al., 2019; Bautista and Amaro, 2023).

Overall, there is a lack of inclusivity of diverse samples within the mindfulness-based intervention literature (Nagy et al., 2022) and while mindfulness is an inherently equitable and accessible practice, it is not delivered equitably across groups (Bautista et al., 2022). The MMWR program is a culturally adapted and tested mindfulness-based intervention with documented efficacy among ethnic and racially diverse women with SUD and PTSD. By continuing to investigate factors that influence mindfulness intervention acceptability, which, in turn, increases retention and improves efficacy, we can create MBIs that are more inclusive and serve a more diverse population.

### Strengths and limitations

The strengths of the present study include a mixed-methods integration of qualitative and quantitative satisfaction data; this integration provides a more complete view of participant experience and satisfaction in the intervention. While satisfaction is commonly measured only at the end of the intervention, our study assessed initial satisfaction, which allowed us to examine initial barriers that subside with additional exposure to the intervention and ongoing or continuous barriers that warrant greater investigation and responsive adaptations in future interventions.

The results from the present study should also be interpreted considering certain limitations. First, to ease participant burden and reduce social desirability bias, participants wrote their qualitative responses, which did not allow for follow-up or clarification questions. Consequently, these write-in responses were not as rich in detail as responses we would expect from one-on-one interviews with the participants. Second, we are not inferring efficacy from the results or using the control group comparison as the control group did not receive exposure to the MMWR program and therefore did not report satisfaction with the program. To see the efficacy results from the MMWR clinical trial, see Black and Amaro (2019). Third, these results may not be generalizable to non-residential treatment facilities, receiving this program while in a residential treatment facility may influence their satisfaction with the program. Fourth, the extreme groups approach allowed us to compare those with the highest trauma symptom severity to those with the lowest trauma symptom severity, which we felt was a meaningful comparison but also limited the inclusion of sample size. The present study is a secondary data analysis; ideally, if a study was focused on assessing differences in satisfaction by trauma symptom severity, they would perform a purposive sampling for those with high- and low-trauma symptom severity. Finally, women with untreated psychiatric disorders were excluded from the study, therefore limiting the generalizability.

### Implications and future suggestions

Overall, this research provides unique insights into the satisfaction of the MMWR program and how the experience with the intervention may differ by trauma symptom severity. Future MBI studies should consider co-occurring SUD and PTSD in their design and delivery of the intervention. This consideration should especially focus on the role of the facilitator and the initial challenge of difficulty focusing reported by women with high-trauma symptom severity.

## Data availability statement

The data analyzed in this study is subject to the following licenses/restrictions: the raw data supporting the conclusions of this article will be made available by the authors, without undue reservation. Requests to access these datasets should be directed to HA hamaro@fiu.edu.

## Ethics statement

The studies involving humans were approved by The University of Southern California Institutional Review Board approved this study (UP-14-00391). All persons gave informed consent prior to their inclusion in the study. The studies were conducted in accordance with the local legislation and institutional requirements. The participants provided their written informed consent to participate in this study.

## Author contributions

TB: Conceptualization, Formal analysis, Methodology, Project administration, Supervision, Validation, Visualization, Writing – original draft, Writing – review & editing. OW: Writing – original draft, Writing – review & editing. MiM: Formal analysis, Writing – review & editing. VR: Formal analysis, Writing – review & editing. YC-C: Formal analysis, Writing – review & editing. AD: Conceptualization, Formal analysis, Writing – review & editing. MaM: Formal analysis, Writing – review & editing. MS: Supervision, Writing – review & editing. HA: Conceptualization, Funding acquisition, Resources, Supervision, Writing – review & editing.
